# An Updated Review on the Genetics of Primary Open Angle Glaucoma

**DOI:** 10.3390/ijms161226135

**Published:** 2015-12-04

**Authors:** Khaled Abu-Amero, Altaf A. Kondkar, Kakarla V. Chalam

**Affiliations:** 1 Glaucoma Research Chair, Department of Ophthalmology, College of Medicine, King Saud University, Riyadh 11424, Saudi Arabia; Khaled.Abu-Amero@jax.ufl.edu (K.A.-A.); akondkar@gmail.com (A.A.K.); 2 Department of Ophthalmology, University of Florida College of Medicine, 580, W, 8th Street, Tower-2, Jacksonville, FL 32209, USA

**Keywords:** epidemiology, genetics, GWAS, POAG, quantitative traits, SNP genotyping

## Abstract

Epidemiological studies suggest that by 2020 the prevalence of primary open angle glaucoma (POAG) is estimated to increase to 76.0 million, and to 111.8 million by 2040 globally due to the population aging. The prevalence of POAG is the highest among those of African descent, followed by Asians, and the lowest in Europeans. POAG is a genetically complex trait with a substantial fraction exhibiting a significant heritability. Less than 10% of POAG cases in the general population are caused by specific gene mutations and the remaining cases are polygenic. Quantitative traits related to POAG pathogenesis such as intra-ocular pressure (IOP), vertical cup/disc ratio (VCDR), optic disc area, and central corneal thickness (CCT) are highly heritable, and likely to be influenced at least in part by genes and show substantial variation in human populations. Recent genome-wide association studies (GWAS) have identified several single nucleotide polymorphisms (SNPs) at different loci including *CAV1/CAV2*, *TMCO1*, *CDKN2B-AS1*, *CDC7-TGFBR3*, *SIX1/SIX6*, *GAS7* and *ATOH7* to be associated with POAG and its related quantitative traits (endophenotypes). The chapter provides a brief overview on the different GWAS and SNP association studies and their correlation with various clinical parameters important for POAG in the population worldwide, including the Middle East.

## 1. Introduction

Glaucoma is a chronic and progressive group of optic neuropathies affecting more than 60 million people globally [1]. It is associated with death of retinal ganglion cells resulting in characteristic cupping or degeneration of the optic nerve head and loss of peripheral vision [2]. Primary open angle glaucoma (POAG) is one of the most common types of glaucoma which is clinically characterized by an open and normal anterior iridocorneal chamber angle [2]. POAG can either occur with increased intraocular pressure (IOP) or normal IOP, the latter being referred to as normal-tension glaucoma (NTG).

Although there are many postulated mechanisms of retinal ganglion cell damage, the exact etiology of POAG still remains obscure. The well-recognized risk factors associated with POAG include elevated IOP, age, family history, gender, ethnicity, central corneal thickness, and myopia. A recent large prospective study indicated that POAG with early paracentral visual field loss displays distinct as well common risk factor profiles as compared to those with peripheral vision loss [3]. Raised IOP is the most important and the only modifiable risk factor in the development and progression of POAG. Several large population-based studies in the past have confirmed that the reduction of IOP reduces the progression of glaucoma in patients with or without elevated IOP [4,5,6,7,8].

Similarly, findings of the meta-analysis from the Eye Diseases Prevalence Research Group have shown that the occurrence of glaucoma increases with increasing age among all ethnicities (Europeans, Blacks, and Hispanics) [7,9]. Age was also reported to be associated with POAG in patients with ocular hypertension in two large population-based studies [5,8]. Family history is another important risk factor in the development of glaucoma [7,10,11,12]. A positive family history of POAG significantly increases the odds (varying from five to 10 times) for the development of POAG [13]. In the Melbourne [14] and Rotterdam studies [11], males showed a trend towards increased risk of POAG which was absent in the Barbados Eye Study [7] and the Beaver Dam Eye Study [10]. Similarly, the Eye Disease Prevalence Research Group [9] reported no gender-related association of glaucoma among the European, African American, and Hispanic subjects. However, a recent systematic review of 3497 POAG cases out of 146,882 participants with gender-specific data showed that the age-adjusted prevalence is higher in men compared to women, and that this finding remains consistent across all ethnic groups provides very strong evidence for the association of POAG with gender [15]. Several studies have shown POAG to be more prevalent with rapid and severe disease progression in people of African-Caribbean as compared to European descent, Hispanics, and Asians [9,16]. Central corneal thickness (CCT) has also been reported to be associated with POAG, particularly in the ocular hypertension patients [17,18]. Although the precise mechanism(s) are still unclear, this may be in part due to the effect of corneal thickness on IOP measurement, and increased susceptibility to optic nerve damage [19,20]. In addition, studies have shown that individuals with thicker corneas are less responsive to topical ocular hypotensive medications [21]. Myopia is also considered to be an important risk factor for POAG as it can increase susceptibility of myopic nerves to glaucomatous damage [22]. Moderate-to-high levels of myopia conferred two- to three-fold increased risk in the Australian [23], US Caucasian [24], and the Chinese populations [25]. Other predisposing factors for POAG include adult-onset diabetes and hypertension. Although there are conflicting reports regarding the risk of POAG in individuals with diabetes [26,27], a recent systematic review and meta-analysis of 13 studies, which included six population-based cohorts and seven case-control studies, showed increased risk of POAG (relative risk of 1.4 and 1.49, respectively) in individuals with diabetes [28]. Multiple epidemiological studies have also reported a role of hypertension as a risk factor for POAG [23,29]. Treatment of hypertensive patients with beta-blockers results in nocturnal hypotension and is a potential risk factor for glaucomatous optic neuropathy [30]. The mechanism(s) by which hypertension induces optic nerve damage are still unclear.

POAG is a genetically complex trait with a substantial fraction exhibiting a significant heritability. Genetic linkage studies of large affected families have so far identified at least 20 chromosomal loci (GLC1A-P) that are linked to POAG. The causative genes that are capable of causing POAG with minimal influence from other gene(s) or the environment and that have been consistently implicated so far include myocilin (*MYOC*), optineurin (*OPTN*), WD repeat domain 36 (*WDR36*), ankyrin repeat and SOCS-box containing 10 (*ASB10*), Cytochrome P450 family 1, subtype B, polypeptide 1 (*CYP1B1*), and neurotrophin 4 (*NTF4*) as reviewed elsewhere [31,32]. Twin studies and family-based studies have discovered a number of genes. However, these disease-causing genes account for <10% of POAG cases in the general population. It is therefore likely that the hereditary aspect of many of the remaining cases of POAG is due to the combined effects of several genes (polygenic) and that gene-environment interactions are important. Quantitative endophenotype traits related to POAG pathogenesis such as IOP, vertical cup-to-disc ratio (VCDR), and CCT [10,33,34] are highly heritable, likely to be influenced at least in part by genes, and are highly polymorphic. Recent advances in genomic technologies and genome-wide association studies (GWAS) have greatly accelerated the discovery and understanding of genes and genomic regions associated with POAG and influencing the quantitative endophenotype traits related to POAG pathogenesis, which will be the main focus of this chapter.

## 2. Epidemiology of POAG

Recent epidemiological studies suggest that, in 2013, almost 64.3 million people (aged between 40 and 80 years) were affected by glaucoma globally, and this number is expected to increase to 76.0 million by 2020 and to 111.8 million by 2040 due to the population aging [35]. POAG accounts for a major three-quarters (74%) of all glaucoma cases [1]. Another recent meta-analysis estimated the global number of POAG cases in 2015 at 57.5 million, rising to 65.5 million by 2020 [15]. Almost half (47%) of these will those of Asian descent, while a quarter (24%) will be European [1]. The risk and subtypes of glaucoma are known to vary among races and countries [36]. A meta-analysis conducted by the Eye Disease Prevalence Research Group showed that, in the United States, African Americans have a higher POAG prevalence than Caucasians. The prevalence of POAG in individuals ≥40 years old was observed to be 1.86%, including 1.57 million Caucasian and 398,000 African American subjects. In 2020, this number is estimated to rise up to 3.36 million due to the population aging [9]. In all the age groups, there was an increased prevalence of glaucoma in individuals of African descent compared with European-derived individuals [37]. Similarly, a recent meta-analysis of 81 studies including 37 countries, 216,214 participants, and 5266 POAG cases reported that the Black populations had the highest POAG prevalence of 5.2% (95% credible interval (CrI) 3.7%, 7.2%) at 60 years, rising to 12.2% (95% CrI 8.9% to 16.6%) at 80 years. The increase in POAG prevalence per decade of age was found to be highest among the Hispanics (2.31, 95% CrI 2.12, 2.52) and Caucasian populations (1.99, 95% CrI 1.86, 2.12), and lowest in East and South Asians (1.48, 95% CrI 1.39, 1.57; 1.56, 95% CrI 1.31, 1.88, respectively). In addition, men were more likely to have POAG than women (1.30, 95% CrI 1.22, 1.41). It is clearly evident that individuals of African descent are associated with increased risk (estimated incidence is two to five times higher) of developing glaucoma compared with individuals of European descent. The reasons for this increased risk of glaucoma among individuals of African descent are still not clear. The Barbados Eye Study reported a prevalence of 7% in Africans, suggesting an influence of ancestral factors [7]. Several other factors that may also be influential could be physiological or anatomical differences in the optic disc or corneas, environmental factors, social differences or genetics [26].

## 3. Genotype-Phenotype Association in POAG

Association studies using the candidate-gene approach and GWASs have been particularly useful tools in identifying genetic factors, each of which may have a relatively small effect but contributes to a large number of cases. Unlike the candidate-gene approach, GWAS is an unbiased (without bias to known protein functionality gene) genome-wide approach that compares the genotypic profile of single nucleotide polymorphisms (SNPs) throughout the genome in cases (affected) and controls (unaffected), thus identifying genomic region(s) associated with a disease or trait of interest. The large population sample required in GWASs to achieve a genome-wide statistical significance (*p*-value of less than 5 × 10^−8^) has been greatly facilitated by the formation of the International Consortia. However, since GWAS can rarely identify functional or causal variant(s), further in-depth genotyping and functional testing in addition to replication studies in independent cohorts of different population groups are considered a standard requirement to conclusively validate genes or genomic regions identified from GWAS. Using this powerful approach (GWAS), recent genetic studies have identified genes or genetic variants with modest effect to be associated with POAG and related quantitative traits (Table 1). These studies have provided better insights into the genetic basis of POAG and improved our understanding of the underlying pathophysiology of the disease.

**Table 1:** Genes and polymorphisms identified in POAG using genome-wide and candidate-gene approaches in the Middle East and other populations.

Studies	Gene/Chromosome	SNP ID	Population *	Study Type	Study Size (POAG/Controls) *	OR/Beta, *p* Value	Any Clinical Association *
**GWAS Studies**							
Nakano *et al.*, 2009 [38]	*PLXDC2 (10p12.31)*	rs7081455	D: Japan R: Japan	GWAS	D: 1519 R: 857	OR = 1.49, *p* = 1 × 10^−5^	–
	*TMTC2 (12q21.31)*	rs7961953				OR = 1.37, *p* = 7 × 10^−5^	–
	*ZP4 (1q43)*	rs547984				OR = 1.34, *p* = 6 × 10^−5^	–
		rs540782				OR =1.34, *p* = 6 × 10^−5^	
		rs693421				OR = 1.35, *p* = 4 × 10^−5^	
		rs2499601				OR = 1.33, *p* = 9 × 10^−5^	
Meguro *et al.*, 2010 [39]	*SRBD1 (2p21)*	rs3213787	Japanese	GWAS	D: 305 R: 355	OR = 2.80, *p* = 2.5 × 10^−9^	Associated with NPG
	*ELOVL5 (6p12.1)*	rs735860	–	–	–	OR = 1.69, *p* = 4.1 × 10^−6^	Associated with NPG
Thorleifsson *et al.*, 2010 [40]	*CAV1/CAV2 (7q31.1)*	rs4236601	D: Iceland R1: SW, UK, AU R2: China	GWAS	D: 36,140 R1: 4239 R2: 879	OR = 1.36, *p* = 5 × 10^−10^	Nominal association was observed for increased IOP (*p* = 0.034)
		rs1052990				OR = 1.32, *p* = 1.1 × 10^−9^	–
Burdon *et al.*, 2011 [41]	*CDKN2B-AS (9p21.3)*	rs4977756	AU, NZ	GWAS	D: 590/3956 R: 4148	OR = 1.50, *p* = 4.7 × 10^−9^	–
	*TMCO1 (1q24)*	rs4656461				OR = 1.68, *p* = 6.1 × 10^−10^	–
Wiggs *et al.*, 2012 [42]	*CDKN2B-AS (9p21)*	rs2157719	US Caucasian	GWAS	D: 3146/3487	OR = 0.69, *p* = 1.86 × 10^−18^	Also associated with NPG. OR = 0.58, *p* = 1.17 × 10^−12^
	*SIX1/SIX6 (14q23)*	rs10483727				OR = 1.32, *p* = 3.87 × 10^−11^	–
	*8q22*	rs284489				OR = 0.62, *p* = 8.88 × 10^−10^	Associated with NPG
Osman *et al.*, 2012 [43]	*CDKN2B-AS (9p21)*	rs1063192	Japanese	GWAS	D: 7993 R: 9014	OR = 0.75, *p* = 5.2 × 10^−11^	–
	*SIX1/SIX6 (14q23)*	rs10483727				OR = 0.79, *p* = 9.49 × 10^−8^	–
	*NCKAP5* *(2q21.2)*	rs7588567				OR = 0.85, *p* = 3.89 × 10^−7^	–
Nakano *et al.*, 2012 [44]	*CDKN2B-AS (9p21.3)*	rs7865618	Japanese	GWAS	D: 833/686 R: 411/289	OR = 1.78, *p* = 9.0 × 10^−11^	Strongly associated with POAG and POAG/NPG, but not with HPG
		rs523096				OR = 1.76, *p* =1.6 × 10^−10^	
Takamoto *et al.*, 2012 [45]	*CDKN2B (9p21)*	rs523096	Japanese	GWAS	D: 286/557 R: 183/514	OR = 2.13, *p* =4.96 × 10^−11^	Associated with NTG
Chen *et al.*, 2014 [46]	*ABCA1 (9q31.1)*	rs2487032	Asian Southern Chinese	GWAS	D: 1007/1009 R: 1899/4965	OR = 0.69, *p* = 1.66 × 10^−8^; OR_R_ = 0.73, *p*_R_ = 2.79 × 10^−9^	–
	*PMM2 (16p13.2)*	rs3785176				OR = 1.42, *p* = 3.18 × 10^−6^; OR_R_ = 1.30, *p*_R_ = 5.77 × 10^−10^	–
Gharahkhani *et al.*, 2014 [47]	*ABCA1 (9q31.1)*	rs2472493	D: Australian R1: Australian R2: US	GWAS	D: 1155/1992 R1: 932/6862 R2: 2616/2634	OR = 1.31, *p* = 2.1 × 10^−19^	–
	*AFAP1 (4p16.1)*	rs4619890				OR = 1.20, *p* = 7.0 × 10^−10^	–
	*GMDS (6p25.3)*	rs11969985				OR = 1.31, *p* = 7.7 × 10^−10^	–
Li *et al.*, 2015 [48]	*CDKN2B-AS1 (9p21)*	rs2157719	Asian, African and European	GWAS	D: 3504/9746 R: 9173/26,780	OR = 0.71, *p* = 2.81 × 10^−33^	–
	*CDC7-TGFBR3 (1p22)*	rs1192415				OR = 1.13, *p* = 1.60 × 10^−8^	Associated with optical disk, vertical CD ratio
	*FNDC3B (3q25.31)*	rs4894796				OR = 0.89, *p* = 7.93 × 10^−8^ in Asians only	–
van Koolwijk *et al.*, 2012 [49]	*GAS7 (17p13.1)*	rs11656696	D: NL R: UK, AU, Canada, NZ SNP: NL, GE	GWAS	D: 11,972 R: 7482 SNP: 1432	*p* = 1.4 × 10^−8^ SNP: P = 2.4 × 10^−2^	Associated with IOP reduction
	*TMCO1 (1q24.1)*	rs7555523				*p* = 1.6 × 10^−8^ SNP: P = 9.1 × 10^−4^	Associated with IOP increase
Hysi *et al.*, 2014 [50]	*FNDC3B (3q25.31)*	rs6445055	Asian, European	GWAS	D: 35,296 R: 4284/95,560	*p* = 4.19 × 10^−8^	All 4 loci associated with IOP
	*ABCA1 (9q31.1)*	rs2472493				*p* = 2.8 × 10^−11^	
	*ABO (9q34.2)*	rs8176693				*p* = 6.39 × 10^−11^	
	*11p11.2*	rs747782				*p* = 1.04 × 10^−11^	
Chen *et al.*, 2015 [51]	*FAR2 (12p11.22)*	rs4931170	US Caucasian	GWAS	D: 1660	*p* = 1.2 × 10^−5^	Associated with IOP
	*GGA3 (17q25.1)*	rs52809447				*p* = 6.7 × 10^−5^	
	*PKDREJ (22q13.31)*	rs7291444				*p* = 7.4 × 10^−5^	
Springelkamp *et al.*, 2015 [52]	*ARHGEF12 (11q23.3)*	rs58073046	D: NL R: NL, UK	GWAS	D: 8105 R: 1125/4117	β = 0.44, *p* = 1.87 × 10^−8^ OR = 1.66, *p* = 2.81 × 10^−9^ (HPG) OR = 1.29, *p* = 4.23 × 10^−2^ (NPG)	Associated with increasing IOP
Ramdas *et al.*, 2010 and 2011 [53,54]	*ATOH7 (10q21.3-22.1)*	rs1900004	D: NL R: NL, UK	GWAS	D: 7360 R: 4455	β = −0.068, *p* = 2.05 × 10^−32^	Optic disc area (−)/VCDR (−)
	*CDC7/TGFBR3 (1p22)*	rs1192415				β = 0.064, *p* = 1.82 × 10^−27^	Optic disc area (+)
	*CDKN2B (9p21)*	rs1063192				β = −0.014, *p* = 1.96 × 10^−14^	VCDR (−)
	*SIX1 (14q22.3-q23)*	rs10483727				β =0.012, *p* = 9.30 × 10^−11^	VCDR (+)
	*SALL1 (16q12.1)*	rs1362756				β = 0.028, *p* = 6.48 × 10^−8^	Optic disc area (+)
Macgregor *et al.*, 2010 [55]	*ATOH7 (10q21.3-22.1)*	rs3858145	D: AU R: UK	GWAS	D: 1368 R: 848	*p* = 3.4 × 10^−10^	Associated with mean disc area
	*RFTN1* *(3p24)*	rs690037				*p* = 1.6 × 10^−6^	Explained 2.1% cup area variation in AU cohort
Khor *et al.*, 2011 [56]	*CARD10 (22q13.1)*	rs9607469	D: Asian R: NL	GWAS	D: 4445 R: 9326	*p* = 2.73 × 10^−12^	Associated with optic disc area
	*ATOH7 (10q21.3-22.1)*	rs7916697				*p* = 2.00 × 10^−15^	Associated with optic disc area in Asians
	*CDC7/TGFBR3 (1p22)*	rs1192415				*p* = 7.57 × 10^−17^	
Iglesias *et al.*, 2014 [57 ]	*SIX6 (14q23)*	rs33912345 (His141Asn)	D: NL, UK R: NL, UK	GWAS	D: 292/1208 R: 11,473	*p* = 7.74 × 10^−7^	Associated with VCDR and POAG
		rs146737847 (Glu29Lys)				*p* = 5.0 × 10^−3^	Associated with VCDR
Vitart *et al.*, 2010 [58]	*COL5A1 9q34.2*	rs1536482	Croatia, Scotland	GWAS	D: 7711 R: 2681	β = 0.22, *p* = 7.1 × 10^−8^	Associated with CCT
	*ZNF469 16q24.2*	rs12447690				β = 0.23, *p* = 4.4 × 10^−9^	
	*AKAP13 15q24-25*	rs6496932				β = 0.13, *p* = 1.4 × 10^−8^	
	*AVGR8 13q12.11*	rs1034200				β = 0.14, *p* = 3.5 × 10^−9^	
Vithana *et al.*, 2011 [59]	*ZNF469 (16q24)*	rs12447690	D1: SG-Malay D2: SG-Chinese	GWAS	D1: 3280 D2: 3400	β = −5.068, *p* = 1.92 × 10^−14^	Associated with CCT
		rs9938149				β = −6.248, *p* = 1.63 × 10^−16^	
	*COL5A1/RXRA (9q34.2-q34.3)*	rs1536478				β = −4.63, *p* = 3.05 × 10^−9^	
		rs7044529				β = 2.7, *p* = 1.2 × 10^−4^	
	*COL8A2 (1p34.2)*	rs96067				β = −4.799, *p* = 5.40 × 10^−13^	
Ulmer *et al.*, 2012 [60]	*ZNF469 (16q24)*	rs12447690	D: US-Cau SNP: US-Cau	GWAS	D: 1117 SNP: 6469	β = −5.08, *p* = 0.001	Associated with CCT
	*NTM (11q25)*	rs7481514				β = −6.89, *p* = 1.03 × 10^−5^	Associated with reduced CCT
						OR = 1.28, *p* = 9.9 × 10^−4^	and POAG risk in low-tension subset
**Candidate Gene Studies**							
Chen *et al.*, 2012 [61]	*2p16.3*	rs1533428	China	SNP	462/577	OR = 2.16, *p* = 0.00025	Associated with late-onset POAG
Kim *et al.*, 2014 [62]	*10p12.31*	rs7098387	Korea	SNP	211/904	OR = 2.0, *p* = 0.00038	Associated with POAG
Fan *et al.*, 2005 [63]	*APOE 19q13.2*	rs429358 rs7412	Japan	SNP	400/281	OR = 0.4, *p* = 0.007	APOE4 confers a protective effect against NTG
Lam *et al.*, 2006 [64]		rs429358 rs7412	China	SNP	400/300	OR = 0.36, *p* = 0.008	APOE4 confers a protective effect against NTG
Lake *et al.*, 2004 [65]		rs429358 rs7412	UK	SNP	155/349	*p* = ns	None
Cao *et al.*, 2012 [66]	*ATOH7 10q21.3-22.1*	rs7916697	African-Caribbean	SNP	272/165	OR = 0.67, *p* = 0.0096	Interacts with rs1063192 near *CDKN2B* to reduce POAG risk
		rs1900004				OR = 1.02, *p* = 0.9076	None
		rs3858145				OR = 0.98, *p* = 0.9138	None
Mabuchi *et al.*, 2012 [67]		rs1900004	Japan	SNP	425/191	*p* = 0.028	Associated with NTG
Chen *et al.*, 2012 [68]		rs3858145	China	SNP	142/289	OR = 2.69, *p* < 0.05	Showed interaction with RFTN1 rs690037
		rs61854782				β = −0.088, *p* = 0.004	Associated with VCDR in controls but not POAG
Fan *et al.*, 2011 [69]		rs1900004	US-Caucasian	SNP	539/336	OR = 1.89, *p* = 0.025	Associated with increased optic nerve area
Dimasi *et al.*, 2012 [70]		rs1900004	AU, NZ	SNP	873/886	OR = 1.12′, *p* = 0.18	No association
		rs3858145				OR = 1.13, *p* = 0.12	
Wiggs *et al.*, 2011 [71]	*CAV1/CAV2 7q31.1*	rs4236601	US-Caucasian	SNP	1000/1183	OR = 1.31, *p* = 0.0007	Significantly associated in women more than men
		rs1052990				OR = 1.25, *p* = 0.0084	Significantly associated in women; and nominally associated with NPG (*p* = 0.039)
Cao *et al.*, 2012 [66]		rs4236601	African-Caribbean	SNP	272/165	OR = 1.15, *p* = 0.3332	No association
Loomis *et al.*, 2014 [72]		rs4236601	US-Caucasian	SNP	R1: 976/2132 R2: 1140/2290	*p*_meta_ = 2.61 × 10^(−7)^, *p*_women_ = 1.59 × 10^(−5)^	Associated with early paracentral VF defect
		rs17588172				*p*_meta_ = 1.07 × 10^(−4)^	Associated with early paracentral VF defect
Kuehn *et al.*, 2011 [73]		rs4236601	US	SNP	545/297	*p* = 0.5	No association
Cao *et al.*, 2012 [66]	*CARD10 22q13.1*	rs9607469	African-Caribbean	SNP	272/165	OR = 1.13, *p* = 0.5096	No association
Cao *et al.*, 2012 [66]	*CDC7/TGFBR3 1p22*	rs1192415	African-Caribbean	SNP	272/165	OR = 1.14, *p* = 0.4802	No association
Dimasi *et al.*, 2012 [70]		rs1192415	AU, NZ	SNP	873/886	OR = 1.22, *p* = 0.03	Showed nominal significance with optic disc area
Cao *et al.*, 2012 [66]	*CDKN2B (-AS1) 9p21*	rs1063192	African-Caribbean	SNP	272/165	OR = 0.39, *p* = 0.0008	Minor allele was protective against POAG
		rs4977756				OR = 0.89, *p* = 0.4507	No association
Fan *et al.*, 2011 [69]		rs1063192	US-Caucasian	SNP	539/336	OR = 0.73, *p* = 0.0006	Associated with decreased VCDR and POAG risk
Mabuchi *et al.*, 2012 [67]		rs1063192	Japan	SNP	425/191	β = 0.11, *p* = 0.0043	Associated with VCDR; and NTG (*p* = 0.023)
Dimasi *et al.*, 2012 [70]		rs1063192	AU, NZ	SNP	873/886	OR = 0.74, *p* = 2.2 × 10^−5^	More strongly associated with advanced open-angle glaucoma
Burdon *et al.*, 2012 [74]		rs10120688 rs7049105	AU, NZ	SNP	1432/595	VCDR – β = 0.016, *p* = 0.03; IOP – β = −2.135, *p* = 0.001	Associated with larger VCDR and lower IOP
Mabuchi *et al.*, 2012 [67]	*CHEK2 22q12.1*	rs1547014	Japan	SNP	425/191	β = 0.11, *p* = 0.0079	Associated with VCDR; and HTG (*p* = 0.013)
Dimasi *et al.*, 2012 [70]		rs1547014	AU, NZ	SNP	873/886	OR = 0.98, *p* = 0.77	No association
Dimasi *et al.*, 2012 [70]	*COL5A1/RXRA 9q34.2-q34.3*	rs1536482	AU, NZ	SNP	873/886	OR = 0.94, *p* = 0.46	No association
		rs7044529				OR = 1.00, *p* = 0.98	
Desronvil *et al.*, 2010 [75]	*COL8A2 1p34.2*	rs274754	US-Caucasian	SNP	100	*p* = 0.018	Associated with corneal thickness
Dimasi *et al.*, 2010 [76]	*FBN1 15q21.1*	rs17352842	AU-Caucasian	SNP	956	*p* = 0.02	Associated with CCT
Rocha *et al.*, 2011 [77]	*GSTT1/GSTM1 1p13.3*	null > positive	Brazil	SNP	87/85	OR = 2.4, *p* = 0.016	T1M0 genotype associated with higher IOP and severe defect of right eye optic nerve and visual field
Juronen *et al.*, 2000 [78]		positive > null	Estonia	SNP	250/202	OR = 1.83, *p* = 0.002	GSTM1 were at significant risk for glaucoma and even higher in smokers (OR = 3.86, *p* = 0.012)
Jansson *et al.*, 2003 [79]		positive = null	Sweden	SNP	200/200	*p* = ns	No association
Fan *et al.*, 2010 [80]		positive = null	China	SNP	405/201	*p* = ns	No association
Liu *et al.*, 2010 [81]	*NTF4 19q13.33*	Whole gene	US-Caucasian	SNP	443/533	–	Not associated with POAG
Rao *et al.*, 2010 [82]		Whole gene	India	SNP	141/285	*p* = 0.2	No association
Vithana *et al.*, 2010 [83]		c.338T>C	China	SNP	174/91	–	Rare cause of POAG in Chinese
Chen *et al.*, 2012 [84]		c.470G>C c.545C>T	China	SNP	720/230	–	May be a rare cause of POAG
Aung *et al.*, 2002 [85]	*OPA1 3q28-q29*	rs166850	UK	SNP	163/86	OR = 3.1, *p* = 0.002	rs166850 combined with rs10451941 was more strongly associated with NTG (*p* = 0.00001)
		rs10451941				*p* = 0.03	
Mabuchi *et al.*, 2007 [86]		rs10451941	Japan	SNP	285/185	OR = 2.27, *p* = 0.004	Increased risk of NTG; and age at diagnosis in HTG (*p* = 0.048)
Yao *et al.*, 2006 [87]		rs166850 rs10451941	African-Caribbean	SNP	109/48	*p* = ns	No association
Fan *et al.*, 2010 [80]		rs166850 rs10451941	China	SNP	405/201	*p* = ns	No association
Dimasi *et al.*, 2010 [76]	*PAX6 11p13*	rs3026398	AU-Caucasian	SNP	956	*p* = 0.02	Associated with CCT; more strongly with rs662702 haplotype (*p* = 0.009)
Chen *et al.*, 2012 [61]	*PLXDC2 10p12.31*	rs7081455	China	SNP	462/577	OR = 1.25, *p* = 0.31	No association
Cao *et al.*, 2012 [66]		rs7081455	African-Caribbean	SNP	272/165	OR = 1.04, *p* = 0.8052	No association
Chen *et al.*, 2012 [68]	*RFTN1 3p24.3*	rs3858145	China	SNP	142/289	β = 25.66, *p* = 0.029	Associated with CCT
Fan *et al.*, 2011 [69]	*SIX1/SIX6 14p22-23*	rs10483727	US-Caucasian	SNP	539/336	OR = 1.33, *p* = 0.0043	Associated with increased VCDR and POAG risk
Dimasi *et al.*, 2012 [70]		rs10483727	AU, NZ	SNP	873/886	OR = 1.38, *p* = 6.2 × 10^−6^	Strongly associated with open-angle glaucoma
Cao *et al.*, 2012 [66]		rs10483727	African-Caribbean	SNP	272/165	OR = 0.77, *p* = 0.4151	No association
Mabuchi *et al.*, 2012 [67]		rs10483727	Japan	SNP	425/191	*p* = 0.017	Associated with age at diagnosis in NTG
Carnes *et al.*, 2014 [88]		rs10483727	US—Caucasians	SNP	262/256	OR = 1.32, *p* = 3.87 × 10^−11^	Significantly associated with POAG
		rs33912345		SNP		OR = 1.27, *p* = 4.2 × 10^−10^	Associated with POAG; and thickness of retinal nerve fiber layer
Mabuchi *et al.*, 2011 [89]	*SRBD1 2p21*	rs3213787	Japan	SNP	370/191	*p* = 0.0003 in NTG and *p* = 0.0013 in HTG	Associated with HTG and NTG including late-onset
Cao *et al.*, 2012 [66]		rs3213787	African-Caribbean	SNP	272/165	OR = 0.45, *p* = 0.2882	None
Takano *et al.*, 2012 [90]	*TLR4 9q33.1*	rs2149356	Japan	SNP	449/107	*p* = 0.000058	Associated with NTG
Chen *et al.*, 2012 [61]		rs7037117	China	SNP	462/577	OR = 0.99, *p* = 0.99	No association
Shibuya *et al.*, 2008 [91]		rs7037117	Japan	SNP	215/318	*p* = 0.0095	1.47- to 1.65-fold increased risk of NTG; strongest association with rs10759930 haplotype
Cao *et al.*, 2012 [66]		rs7037117	African-Caribbean	SNP	272/165	OR = 0.73, *p* = 0.0571	No association
Sharma *et al.*, 2012 [92]	*TMCO1 1q24*	rs4656461	AU, NZ	SNP	1420	β = −2.56, *p* = 0.004	Correlation with age at diagnosis
Ozel *et al.*, 2014 [93]		rs7518099	US-Caucasian	SNP	6.236	*p* = 8 × 10^−8^	Strongly associated with IOP
Chen *et al.*, 2012 [61]	*TMTC2 12q21.31*	rs7961953	China	SNP	462/577	OR = 1.15, *p* = 0.35	No association
Cao *et al.*, 2012 [66]		rs7961953	African-Caribbean	SNP	272/165	OR = 0.89, *p* = 0.5559	No association
Fan *et al.*, 2010 [80]	*TNF*α *6p21.3*	rs1800629	China	SNP	405/201	*p* = 0.012	Associated with HTG
Wang *et al.*, 2012 [94]		rs4645836	China	SNP	234/230	OR = 0.63, *p* = 0.017	Protective for POAG
Mossböck *et al.*, 2006 [95]		rs1800629	AU	SNP	114/228	OR = 0.96, *p* > 0.05	Not associated among Caucasian
		rs361525				OR = 0.52, *p* > 0.05	
Rao *et al.*, 2010 [82]	*VAV2 9q34.1*	rs2156323	India	SNP	141/285	*p* = 0.533	No association
	*VAV3 1p13.3*	rs2801219				*p* = 0.133	
Dimasi *et al.*, 2012 [70]	*ZNF469 16q24*	rs12447690	AU, NZ	SNP	873/886	OR = 1.01, *p* = 0.91	No association
		rs9938149				OR = 0.94, *p* = 0.46	
Chen *et al.*, 2012 [61]	*ZP4 1q43*	rs693421	China	SNP	462/577	OR = 0.98, *p* = 0.31	No association
Cao *et al.*, 2012 [66]		rs547984	African-Caribbean	SNP	272/165	OR = 1.05, *p* = 0.7374	No association
Kim *et al.*, 2014 [62]		rs693421	Korea	SNP	211/904	OR = 1.4, *p* = 0.0082	Associated with POAG
Li *et al.*, 2015 [48]	*CDKN2B-AS1 9p21*	rs2157719	Saudi Arabia	SNP	R **: 236/655	OR = 1.24, *p* = 0.146	–
	*CDC7-TGFBR3 1p22*	rs1192415				OR = 1.24, *p* = 0.146	–
	*FNDC3B 3q25.31*	rs4894796				OR = 1.03, *p* = 0.779	–
Neamatzadeh *et al.*, 2015 [96]	*TP53 17p13.1*	rs1042522	Iranian	SNP	65/65	OR = 2.1, *p* < 0.05	Pro72 allele is associated with POAG risk
Emam *et al.*, 2014 [97]	*NOS3 7q36*	rs2070744	Egypt	SNP	160/110	OR = 1.86, *p* < 0.0001	rs2070744 is associated with high tension glaucoma; and with plasma nitrite/nitrate levels (*p* < 0.001)
		rs1799983			–	OR = 1.28, *p* = 0.21	–
		27 bp-VNTR-a/b			–	OR = 0.81, *p* = 0.33	–
Abu-Amero *et al.*, 2013 [98]	*CAT 11p13*	rs1001179	Saudi Arabia	SNP	225/403	OR = 0.81, *p* = 0.218	Associated with age of onset, and trend towards IOP, and duration of glaucoma
Abu-Amero *et al.*, 2014 [99]	*SOD2 6q25.3*	rs4880	Saudi Arabia	SNP	226/403	OR = 1.0, *p* = 0.988	Trend towards age of onset and IOP
Abu-Amero *et al.*, 2012 [100]	*CAV1/CAV2 7q31*	rs4236601	Saudi Arabia	SNP	220/405	OR = 1.06, *p* = 0.699	–
Abu-Amero *et al.*, 2012 [101]	*LOXL1 15q24.1*	rs1048661	Saudi Arabia	SNP	96/101	*p* = 0.866	–
		rs3825942			–	*p* = 0.477	–
		rs2165241			–	*p* = 0.176	–
Abu-Amero *et al.*, 2006 [102]	*MYOC 1q24.3*	22259 G/T (G324V)	Saudi Arabia	SNP	27/96	*p* = 0.74	–
	*OPTN 10p13*	412 G/A (T34T)			–	*p* = 0.61	–
		469 G/C (Q53H)			–	*p* = 0.28	–
Zanon-Moreno *et al.*, 2013 [103]	*SLC23A2 20p13*	rs1279683	Mediterranean	SNP	250/250	OR = 2.47, *p* < 0.001	Associated with POAG risk; and plasma vitamin C levels (*p* < 0.001)
	*TTPA 8q12.3*	rs6994076			–	OR = 1.38, *p* = 0.122	Associated with plasma vitamin E levels (*p* < 0.001)
	*SEC14L2/TAP 22q12.2*	rs737723			–	OR = 2.24, *p* < 0.001	Associated with POAG risk; and nominal (*p* = 0.047) gene-gene interaction with SNP rs1279683
	*GPX4 19p13.3*	rs757228			–	OR = 0.80, *p* = 0.337	–
Zanon-Moreno *et al.*, 2011 [104]	*RBP1 3q23*	rs176990	Mediterranean	SNP	150/150	OR = 0.97, *p* = 0.826	–
		rs190910			–	OR = 0.83, *p* = 0.315	–
	*SLC23A1 5q31.2*	rs10063949			–	OR = 1.19, *p* = 0.552	–
	*SLC23A2 20p13*	rs1279683			–	OR = 1.67, *p* = 0.010	Associated with POAG risk; and plasma vitamin C levels (*p* < 0.001)
Abu-Amero *et al.*, 2008 [105]	*GSTT1/GSTM1 1p13.3*	T0M0	Saudi Arabia	SNP	49/120	OR = 5.67, *p* = 0.06	GSTT1 and GSTM1 positive genotypes are at risk for POAG
		T1M0			–	OR = 10.2, *p* = 0.00001	–
		T0M1			–	OR = 11.3, *p* = 0.00001	–
Unal *et al.*, 2007 [106]	*GSTT1/GSTM1 1p13.3*	T0M1	Turkey	SNP	144/121	OR = 3.46, *p* < 0.005	GSTM1 positive and GSTT1 null genotypes are associated with increased risk of POAG
Al-Dabbagh *et al.* [107]	*APOE 19q13.2*	rs429358 rs7412	Saudi Arabia	SNP	60/130	OR = 2.75, *p* = 0.034	*APOE4* allele is a risk factor for POAG
Saglar *et al.*, 2009 [108]	*APOE 19q13.2*	rs429358 rs7412	Turkey	SNP	75/119	*p* = 0.38	–
	*TP53 17p*	rs1042522			–	*p* = 0.12	–
Nilforoushan *et al.* [109]	*MTHFR 1p36.3*	rs1801133	Iran	–	73/90	*p* = 0.337	–

* AU—Australia; CCT—central corneal thickness; D—discovery cohort; GE—Germany; HPG—high-pressure glaucoma; HTG—high-tension glaucoma; IOP—intraocular pressure; POAG—primary open angle glaucoma; NPG—normal-pressure glaucoma; NTG—normal-tension glaucoma; NL—Netherland; NZ—New Zealand; R—replication cohort; SG—Singapore; SW—Sweden; UK—United Kingdom; US—United States; VCDR—vertical cup-to-disc ratio. ** Part of an International Glaucoma Genetics Consortium Replication Study.

## 4. GWAS and POAG

Nakano *et al.* described the first GWAS in the Japanese POAG population with patients predominantly having NTG [38]. This was a two-stage GWAS involving a discovery cohort and a replication cohort. The study reported significant loci on chromosomes 1, 10 and 12 that included genes such as *ZP4*, *PLXDC2* and *TMCT2* (*DKFZp762A217*), respectively. However, none of the SNPs achieved a genome-wide significance (*p* < 5 × 10^−8^) even in the combined analysis and, therefore, they await further evaluation in additional cohorts. Meguro *et al.* reported the first genome-wide significant (*p* = 2.5 × 10^−9^, odds ratio (OR) = 2.80) association for SNP rs3213787 in *SRBD1* in the Japanese NTG population [39]. Two other studies have replicated this finding in a Japanese NTG and high-tension glaucoma (HTG) cohort [89] and a US Caucasian POAG cohort [110], but not in the African-Caribbean cohort [66].

GWASs have been able to identify certain common variants that are of significance to the understanding of POAG pathogenesis. These include SNPs near *CAV1* and *CAV2* in an Icelandic cohort [40], in *TMCO1* and *CDKN2B*-*AS1* in an Australian cohort [41], in *CDKN2B-AS1*, *SIX1/SIX6*, and the 8q22 locus in Europeans [42], in *GAS7* and *TMCO1* in US Caucasians [49], and in *CDKN2B-AS1*, *CDC7/TGFBR3* and *FNDC3B* in Asian, African and European cohorts [48].

The *caveolin* genes have been postulated to influence transforming growth factor-beta (TGF-β) or nitric oxide signaling pathways involved in POAG pathogenesis. The locus on chromosome 7q31 has been studies in US Caucasians, Africans, and the Saudi Arabian population with inconsistent results [40,66,71,72,73,100]. A recent meta-analysis of five studies, including 5774 POAG cases and 40,598 healthy controls, suggested that SNP rs4236601 is associated with POAG risk in Caucasian and Asian populations but not in African and Saudi populations [111]. Australian GWAS identified two loci, *TMCO1* (1q24) and *CDKN2B*-*AS1* (9p21), to be associated with advanced glaucoma. The association of the *TMCO1* locus with POAG has been replicated in another GWAS for a Caucasian cohort [49], and associated with increase in IOP as well [49,93]; the carriers of risk alleles for SNP rs4656461 have been reported to be associated with a younger age at diagnosis [92]. The ciliary body, trabecular meshwork and retina show abundant TMCO1 expression. However, its precise role in POAG pathogenesis is unclear. So far, there are no published reports of association studies at the *TMCO1* locus in the Middle East population.

Since the identification of the association between the *CDKN2B/CDKN2B-AS1* locus and POAG in the Australian cohort, several GWASs have replicated this association in the US Caucasian [42], Japanese [43,44,45], Asian, African, and European populations [48], providing strong evidence for the association of this locus with POAG. In addition, many studies have reported a positive association of SNPs in *CDKN2B* in several other populations using a candidate-gene approach [66,67,69,70,74]. These SNPs are located in an anti-sense non-protein coding gene, *CDKN2BAS*, within the *CDKN2A*/*B* gene cluster. CDKN2B is a tumor suppressor gene and, with its suggested role in the TGF-β pathway, may play a critical role in glaucoma pathogenesis [112,113]. Interestingly, carriers of the *CDKN2B-AS1* risk alleles are associated with larger VCDR [53,54] and low IOP as compared to the wild-type carriers [74]. On the basis of these findings, it has been suggested that the *CDKN2B/CDKN2B-AS1* locus of 9p21 may possibly predispose a person to glaucomatous optic neuropathy in a mechanism that may not be dependent on IOP and highlights the importance of the chromosome 9p21 susceptibility locus as a risk factor in the development of POAG [114].

Recently, Li *et al.* performed a GWAS on 3504 POAG cases and 9746 controls. The positive significant findings of this phase were then replicated in 9173 POAG cases and 26,780 controls across 18 different collections of Asian, African, and European populations including a replication cohort from our center in Saudi Arabia [48]. The study confirmed and provided strong evidence of an association at the *CDKN2B-AS1* locus (rs2157719, OR = 0.71, *p* = 2.81 × 10^−33^), and also identified SNP rs1192415 in the *CDC7-TGFBR3* gene (1p22) showing significant association with POAG (OR = 1.13, *p* = 1.60 × 10^−8^) in the Asian, African and European populations, as well as SNP rs4894796 in *FNDC3B* (3q25.31) showing a significant association in Asians only (OR = 0.89, *p* = 7.93 × 10^−8^). Interestingly, these results were found to be non-significant in the Saudi replication cohort, indicating that the genetic cause for POAG in the Saudi population may be different than those from Asian, African and European descent.

GWAS studies by Wiggs *et al.* and Osman *et al.* in the Caucasian POAG and Japanese POAG cases, respectively, have demonstrated a strong association of SNP rs10483727 located in the in the intergenic region between the *SIX1* and *SIX6* locus (14q23) [42,43]. SIX6 has been shown to express in the developing and adult human retina [115]. Moreover, the association of SNP rs10483727 in the *SIX1*/*SIX6* region has also been replicated in other Caucasian POAG cohorts [67,69,70,88] but not in the African-Caribbean subjects [66]. After the association of the *CDKN2B-AS1* region on chromosome 9p21, the second most consistent association with POAG has been observed in the *SIX1/SIX6* locus and so it would be interesting to know if this locus is associated with POAG in the Saudi or other Middle Eastern populations. However, currently there are no published reports of association of *SIX1*/*SIX6* locus with POAG in the middle-east population.

Recently, *11p11.2* (containing multiple genes), *ABCA1*, *ABO*, *AFAP1*, *ARHGEF12*, *FAR2*, *GGA3*, *GMDS*, *PKDREJ*, and *PMM2* were added to the newly discovered genes associated with POAG [46,47,50,51,52]. These variants were significantly associated with glaucoma and the related functional visual field loss that could make them future study targets for glaucoma patients in the Middle East.

## 5. GWAS and Quantitative Endophenotype Traits

The genetic evaluation of quantitative endophenotype traits is often very useful in complex multifactorial diseases to understand the contribution of specific traits to the overall disease phenotype. A similar strategy has been successfully used in POAG to understand the contribution of proposed endophenotypes including IOP, VCDR, optic disc area and CCT to the overall disease process. GWASs have been performed to examine the genetic components of these endophenotypes in POAG and the normal population. van Koolwijk and colleagues performed a GWAS for IOP in POAG patients of European descent and identified SNPs rs11656696 and rs7555523, located in *GAS7* and *TMCO1*, respectively, suggesting a role for these two genes in IOP regulation [49]. Other loci found to be associated with IOP so far include *FNDC3B*, *ABCA1*, *ABO*, *11p11.2*, *ARHGEF12* [50,52]. Another three loci, *FAR2*, *GGA3*, and *PKDREJ*, did not reach a genome-wide significance level (*p* < 10^−5^) [51]. Three independent GWASs have evaluated the association of optic disc parameters (VCDR and optic disc area) in the normal general population. The loci associated included *ATOH7*, *CDC7/TGFBR3* and *SALL1*, *CARD10* for the optic disc area, and *CDKN2B*, *SIX1*, *SCYL1*/*LTBP3*, *CHEK2*, and *DCLK1*, in addition to *ATOH7*, for the VCDR [53,54,55,56]. An exome sequencing also reported the *SIX6* locus to influence VCDR (*p* = 7.74 × 10^−7^) [57]. A subsequent meta-analysis of the Rotterdam study with the Twin UK study [54] demonstrated a strong association of *ATOH7*, *CDKN2B*, and *SIX1* in POAG with borderline association for *CDC7*/*TGFBR3* and *SALL4* (both *p* = 0.04). *CARD10* was not found to be associated with African-Caribbean POAG cases [66], whereas *CHEK2* was reported to be associated with VCDR and HTG among the Japanese [67] but not in Europeans [70]. Moreover, multiple studies have provided strong evidence of association of *ATOH7* [66,67,68,69] *CDKN2B*(-*AS1*) [66,67,69,70,74] and *SIX1*/*SIX6* [69,70] with POAG. CCT is an important risk factor for POAG in individuals with increased IOP, and over 26 loci have been reported [116]. GWASs have identified several loci associated with CCT in the normal general population (Asian and European descent) and POAG cases (US Caucasians). These loci include *ZNF469*, *COL5A1*, *AKAP13*, *AVGR8*, and *COL8A2* [58,59,60]. The *ZNF469* and *COL5A1* loci have been found to be associated with CCT in both the Caucasian and Asian cohorts [58,59].

The possible role of these newly discovered loci associated with POAG and its endophenotypes in understanding the pathophysiology of POAG has been elegantly reviewed by Iglesias *et al.* elsewhere [117]. The review integrates current knowledge in POAG from human and experimental data and dissects the contribution of the newly discovered genetic loci with the known molecular and biological processes, including extracellular matrix remodeling; TGF-β and tumor necrosis factor α (TNF-α) signaling; and the vascular tone pathway, that have been implicated in the pathogenesis of POAG.

## 6. Candidate Genes and POAG

Recent reviews by Takamota and Araie [32] and Janssen *et al.* [31] presented a list of genes identified from numerous GWAS and association studies thus far. Taken together, the list of almost 50 genes may represent highly likely candidate genes that may be involved in POAG pathogenesis. Many studies have been performed to replicate the GWAS findings in the Asian, African-Caribbean and Caucasian/European populations using the candidate-gene approach [61,66,67,69,70,71,72,73,74,75,76,84,89,92,93]. Also, many studies were performed to test the association of specific known genes/SNPs with POAG using the same approach in different populations including Middle Eastern [61,62,63,64,65,66,76,77,78,79,80,81,82,83,84,85,86,87,90,91,94,95,96,97,98,99,100,101,102,103,104,105,106,107,108,109]. These SNP replication and genetic association studies in the Middle Eastern and other populations are also listed in Table 1. Among these, consistent findings have been reported for *ATOH7* [66,67,68,69], *CDKN2B*(-*AS1*) [66,67,69,70,74], *GSTT1*/*GSTM1* [77,78,105,106], *SIX1*/*SIX6* [69,70] and *TMCO1* [92,93] loci, indicating a potential role of these genes/loci in the pathogenesis of POAG. However, except for the glutathione-S transferase (GST) polymorphism, none of these loci have been either found to be associated with POAG (e.g., *CAV1*/*CAV2*, *CDC7*/*TGFBR3*, *FNDC3*) or the association has not been reported yet (e.g., *ATOH7*, *CDKN2B*(-*AS1*), *SIX1*/*SIX6*, *TMCO1*) in the Middle Eastern population. However, the positive findings of *GSTT1* and *GSTM1* genotypes in the Middle Eastern population may be very interesting, highlighting the role of anti-oxidants and/or oxidative stress-related pathways/mechanisms in the pathogenesis of POAG in this population. This view is strongly supported by recent meta-analysis studies that examined the association of GST polymorphisms and the risk of POAG [118,119,120]. We have previously studied SNPs in two of the anti-oxidant genes, *CAT* (rs1001179) and *SOD2* (rs4880) [98,99], in the Saudi POAG patients. However, the studies did not provide any direct association with POAG but indicated a trend towards an association with IOP and age of onset of POAG. In addition, some studies have demonstrated moderate evidence for association of SNPs in *TP53*, *NOS3*, *SEC14L2*/*TAP*, and *APOE* [96,97,103,107]. However, these studies have been limited by sample size and would need further investigations in a large population-based cohort. The examination of causative genes such as *MYOC*, *OPTN* and *LOXL1* in Saudi POAG cases has also provided negative results [102,105]. Table 2 list all genes associated with POAG and their possible role in POAG pathogenesis.

## 7. Final Remarks

There is significant progress in understanding the genetic basis of POAG, largely due to the application of GWAS methodology in different populations. In recent years, GWASs have identified several loci associated with POAG including *CAV1/CAV2*, *TMCO1*, *CDKN2B-AS1*, *CDC7-TGFBR3*, *SIX1/SIX6*, *GAS7* and *ATOH7*.

The association between the *CDKN2B*(*-AS1*) locus on chromosome 9p21 and POAG has been extensively established across different populations and represents a major genetic risk factor for POAG. Studies involving the *SIX1/SIX6* and the *ATOH7* loci affecting the optic disc parameters and POAG itself have also been reproducible. Other loci seem to be more ethnicity-specific. *CAV1*/*CAV2* and *CDC7*-*TGFBR3* loci do not seem to contribute to POAG in the Middle East and the role of other newly discovered loci is yet to be established. Moreover, the *GSTT1*/*GSTM1* genotypes were found to be strongly associated with POAG in the Middle Eastern population and more studies may be needed to examine the role of oxidative stress and anti-oxidant pathways in this population.

Based on the current and new genes identified in glaucoma, it may be possible to develop an algorithm of SNP risk scores to assess the future risk of POAG in patients, which could be clinically useful. However, despite the tremendous progress, the genetic basis of POAG is still not completely understood and further investigations are needed to identify novel genes and pathways contributing to glaucoma that may help define disease-specific targets and facilitate the development of diagnostic and therapeutic strategies.

**Table 2:** Possible pathogenesis role of various genes associated with POAG.

Gene	Gene Name	Function	Role in Ophthalmic Diseases
*PLXDC2*	Plexin Domain Containing 2	May play a role in tumor angiogenesis	Possible role through inhibition of angiogenesis and possible involvement in protecting against inflammation
*TMTC2*	Transmembrane and Tetratricopeptide Repeat Containing 2	Protein binding calcium ion homeostasis	Unknown
*ZP4*	Zona Pellucida Glycoprotein 4	Signal transducer activity	Unknown
*SRBD1*	S1 RNA Binding Domain 1	Nucleic acid binding, RNA binding, hydrolase activity, acting on ester bonds	Appears to contribute to glaucomatous optic neuropathy as a non–IOP-related genetic factor; exact mechanism is not known
*ELOVL5*	ELOVL Fatty Acid Elongase 5	Catalytic activity	Appears to contribute to glaucomatous optic neuropathy as a non–IOP-related genetic factor; exact mechanism is not known
*CAV1/CAV2*	Caveolin 1/Caveolin 2	Receptor binding, structural molecule activity	Dysfunction of cellular signaling and transport leading to the damage in tissues
*CDKN2B-AS*	Cyclin-Dependent Kinase Inhibitor 2B	Protein coding gene, inhibits CDK4	Associated with systemic diseases inside and outside the eyes causing disruption in cell cycle
*TMCO1*	Transmembrane And Coiled-Coil Domains 1	Encoding transmembrane protein	Association with cellular malfunction and oxidative stress
*SIX1*	SIX Homeobox 1	Regulation of cell proliferation, apoptosis and embryonic development.	Associated with developmental malformation of anterior angle, TM and CB
*NCKAP5*	NCK-Associated Protein 5	Protein coding gene	Unknown
*ABCA1*	ATP-Binding Cassette, Sub-Family A (ABC1), Member 1	Cholesterol carrying out of the cell	Expressed highly in TM network, thought to be involved in raising IOP
*AFAP1*	Actin Filament Associated Protein 1	signaling pathways	Possible involvement in aqueous outflow and IOP
*GMDS*	GDP-Mannose 4,6-Dehydratase	Catalytic activity	*GMDS* encodes a protein that is required for the first step in *de novo* synthesis of fucose. Fucose is required for diverse biological functions such as growth factor receptor signalling. Several studies have suggested the effects of growth factors on development of glaucoma
*CDC7*	Cell Division Cycle 7	Phosphorylation	Impairment of cellular function in CB, TM and RGC
*FNDC3B*	Fibronectin Type III Domain Containing 3B	Poly(A) RNA binding	Associated with IOP through as yet unknown mechanism
*GAS7*	Growth Arrest-Specific 7	Protein coding gene sequence-specific DNA binding transcription factor activity	Involved in developmental and functional impairment of RGC
*ABO*	ABO Blood Group (Transferase A, Alpha 1-3-*N*-Acetylgalactosaminyltransferase; Transferase B, Alpha 1-3-Galactosyltransferase)	Basis of the ABO blood group system	Thought to play a role in IOP elevation; Exact mechanism is not known
*FAR2*	Fatty Acyl CoA Reductase 2	Catalytic activity	Unknown
*GGA3*	Golgi-Associated, Gamma Adaptin Ear Containing, ARF Binding Protein 3	Protein sorting and trafficking between the trans-Golgi network (TGN) and endosomes	Unknown
*PKDREJ*	Polycystin (PKD) Family Receptor For Egg Jelly	May have a central role in fertilization	Elevated IOP through undetermined mechanism
*ARHGEF12*	Rho Guanine Nucleotide Exchange Factor (GEF) 12	May play a role in the regulation of RhoA GTPase	Elevated IOP through undetermined mechanism
*ATOH7*	Atonal Homolog 7	Involved in the differentiation of retinal ganglion cells	Involved in developmental problems of retinal vasculature
*SALL1*	Spalt-Like Transcription Factor 1	Organogenesis	SALL1 is involved in development of calcium homeostasis in the endoplasmic reticulum
*RFTN1*	Raftlin, Lipid Raft Linker 1	Formation and/or maintenance of lipid rafts.	Related to vertical cup-to-disc ratio
*CARD10*	Caspase Recruitment Domain Family, Member 10	Protein binding, receptor signaling	Developmental problems of neuronal tissues
*COL5A1*	Collagen, Type V, Alpha 1	Fibril formation	Associated with malformation of connective tissues leading to problems in cornea and TM
*ZNF469*	Zinc Finger Protein 469	Transcriptional regulation	Thought to be involved in central corneal thickness
*AKAP13*	A Kinase (PRKA) Anchor Protein 13	Protein binding, cAMP-dependent protein kinase activity	Involvement in corneal thickness and disruptions in signaling pathways in CB, TM and RGCs
*COL8A2*	Collagen, Type VIII, Alpha 2	Protein binding, extracellular matrix structural constituent	Associated with malformation of connective tissues leading to problems in cornea and TM
*NTM*	Neurotrimin	Protein binding	Unknown
*APOE*	Apolipoprotein E	Protein binding, receptor binding	Role in oxidative stress and disrupted cellular homeostasis in CB, TM, LC and RGC
*CHEK2*	Checkpoint Kinase 2	Protein kinase activity	High expression is associated with problems in optic nerve and cup disk ratio
*FBN1*	Fibrillin 1	Extracellular matrix structural constituent	Mutations in FBN1 could cause backward bowing by compromising the mechanical properties of the iris
*GSTT1*	Glutathione S-Transferase Theta 1	Glutathione transferase activity	Oxidative stress in all the POAG-involved tissues
*NTF4*	Neurotrophin 4	Protein binding, receptor binding	Retinal ganglion cells survival and apoptosis
*OPA1*	Optic Atrophy 1	Protein binding	Involved in Oxidative stress in cornea, CB and TM
*PAX6*	Paired Box 6	Sequence-specific DNA binding RNA polymerase II transcription factor activity	Developmental impairment of neuro ophthalmic system
*PLXDC2*	Plexin Domain Containing 2	Receptor binding	Developmental problems leading to fewer retinal ganglion cells
*SIX6*	SIX Homeobox 6	DNA binding, protein binding	Associated with developmental malformation of anterior angle, TM and CB
*TLR4*	Toll-Like Receptor 4	Receptor binding	Involved in Oxidative stress and decreased cellular viability
*TMTC2*	Transmembrane And Tetratricopeptide Repeat Containing 2	Identical protein binding	TMTC2 is implicated in calcium homeostasis in the endoplasmic reticulum
*TNF*α	Tumor Necrosis Factor	Protease binding, cytokine activity	May be activated in reaction to POAG-related indices (increased IOP, oxidative stress and increase in disregulation of cellular homeostasis
*VAV2*	Vav 2 Guanine Nucleotide Exchange Factor	Epidermal growth factor receptor binding	Unknown
*LOXL1*	Lysyl Oxidase-Like 1	Copper ion binding	Through the loss of elastin formation and resulting friction between the iris and the anterior lens capsule
*ZNF469*	Zinc Finger Protein 469	DNA binding	Associated with developmental malformation of connective tissues leading to problems in cornea and TM
*Zp4*	Zona Pellucida Glycoprotein 4	Signal transducer activity	Unknown
*TP53*	Tumor Protein P53	Core promoter sequence-specific DNA binding	Unknown
*NOS3*	Nitric Oxide Synthase 3 (Endothelial Cell)	Receptor binding	Dysregulation of the vascular tone particularly through interaction with endothelial nitric oxide synthase and production of nitric oxide (NO) in the vascular endothelia. This may lead to decreased AH outflow and increased IOP
*CAT*	Catalase	Catalytic activity	Detoxification of reactive oxygen species—linked to POAG through oxidative stress
*SOD2*	Superoxide Dismutase 2, Mitochondrial	Oxygen binding, DNA binding	Possible role through oxidative stress mechanism
*OPTN*	Optineurin	Protein binding	Through oxidative stress/the mitochondrial caspase-dependent cell death
*TTPA*	Tocopherol (Alpha) Transfer Protein	Transporter activity	Linked to vitamin C loss and that in turn is linked to POAG development through yet undiscovered mechanism
*RBP1*	Retinol Binding Protein 1, Cellular	Transporter activity, retinoid binding	Through retinol and oxidative stress mechanism
*MTHFR*	Methylenetetrahydrofolate Reductase (NAD(P)H)	Methylenetetrahydrofolate reductase (NAD(P)H) activity	Linked through homocysteine level, link to POAG is not established
*GPX4*	Glutathione Peroxidase 4	Glutathione peroxidase activity	Effect on decreased level of vitamins E and C. Lower level of vitamin C is linked to glaucoma through unknown mechanism.
*SEC14L2*	SEC14-Like 2 (*S. Cerevisiae*)	Phospholipid binding	Effect on decreased level of vitamin C. Lower level of vitamin C is linked to glaucoma through unknown mechanism
*SLC23A1*	Solute Carrier Family 23 (Ascorbic Acid Transporter), Member 1	Nucleobase transmembrane transporter activity	Effect on decreased level of vitamin C. Lower level of vitamin C is linked to glaucoma through unknown mechanism
*PMM2*	Phosphomannomutase 2	Catalytic activity	Expressed highly in TM network, thought to be involved in raising IOP
*SLC23A2*	Solute Carrier Family 23 (Ascorbic Acid Transporter), Member 2	Nucleobase transmembrane transporter activity	May be through lowering the plasma level of vitamin C. Low level of vitamin C was found in POAG patients carrying mutation in this gene. Exact link between low vitamin C level and POAG is not determined

Since the advent of GWAS studies, more and more genes and SNPs have been discovered in association of POAG. However, the usefulness (in term of clinical application and developing therapeutic modalities) of those discoveries is still limited. It will take multiple genotype-phenotype studies in various centers and multiethnic groups before establishing the applicability of those SNPs and/or genes to POAG or POAG clinical indices. As for the development of new therapeutic agents, the process will be lengthy and may take several years before effective therapeutic modalities for POAG are available. The whole process from discovering new genetic markers (SNPs) or genes to developing new therapeutic agents may take several steps and many years. Those steps are: (i) Discover those genes and/or SNPs associated with POAG, which is underway thanks to new emerging technologies in molecular genetics such as exome sequencing and GWAS technologies. This may take up to 10 years to complete; (ii) Establish the association of various SNPs and genes with POAG in various ethnicities, larger cohorts, and in multiple centers. This is important as initial discovery studies are conducted on specific ethnicities and in smaller cohorts; (iii) Conduct functional studies in order to understand how those genes and/or SNPs contribute to POAG pathogenesis; (iv) Develop therapeutic agents based on our understanding of the function of the genes associated with POAG. This step is the longest and expected to take at least 10–15 years. This should not hold us back or make us think less of genetic studies as those may prove to be the only way to improve our current understanding of the etiology of glaucoma and facilitate the development of diagnostic and therapeutic strategies.
